# Long-Chain Acylcarnitines and Monounsaturated Fatty Acids Discriminate Heart Failure Patients According to Pulmonary Hypertension Status

**DOI:** 10.3390/metabo11040196

**Published:** 2021-03-26

**Authors:** Maxime Tremblay-Gravel, Annik Fortier, Cantin Baron, Chloé David, Pamela Mehanna, Anique Ducharme, Julie Hussin, Qinghua Hu, Jean-Claude Tardif, Christine Des Rosiers, Jocelyn Dupuis, Matthieu Ruiz

**Affiliations:** 1 Montreal Heart Institute, Université de Montréal, Montréal, QC H1T1C8, Canada; maxime.tremblay-gravel@umontreal.ca; 2 Montreal Health Innovations Coordinating Center Institute, Montréal, QC H1T1C8, Canada; annik.fortier@mhicc.org; 3 Research Center, Montreal Heart Institute, Montréal, QC H1T1C8, Canada; cantin.baron@hotmail.fr (C.B.); chloe.david@umontreal.ca (C.D.); pamela.mehanna@gmail.com (P.M.); anique.ducharme@icm-mhi.org (A.D.); julie.hussin@umontreal.ca (J.H.); Jean-Claude.Tardif@icm-mhi.org (J.-C.T.); christine.des.rosiers@mhi-rc.org (C.D.R.); dupuisj@icloud.com (J.D.); 4 Department of Medicine, Université de Montréal, Montréal, QC H3T1J4, Canada; 5 Department of Pathophysiology, School of Basic Medicine, Tongji Medical College, Huazhong University of Science and Technology, Wuhan 430030, China; qinghuaa@mails.tjmu.edu.cn; 6 Key Laboratory of Pulmonary Diseases of Ministry of Health, Tongji Medical College, Huazhong University of Science and Technology, Wuhan 430030, China; 7 Department of Nutrition, Université de Montréal, Montréal, QC H3T1J4, Canada

**Keywords:** acylcarnitines, fatty acids, heart failure, type 2 pulmonary hypertension

## Abstract

Defects in fatty acid (FA) utilization have been well described in group 1 pulmonary hypertension (PH) and in heart failure (HF), yet poorly studied in group 2 PH. This study was to assess whether the metabolomic profile of patients with pulmonary hypertension (PH) due HF, classified as group 2 PH, differs from those without PH. We conducted a proof-of-principle cross-sectional analysis of 60 patients with chronic HF with reduced ejection fraction and 72 healthy controls in which the circulating level of 71 energy-related metabolites was measured using various methods. Echocardiography was used to classify HF patients as noPH-HF (*n* = 27; mean pulmonary artery pressure [mPAP] 21 mmHg) and PH-HF (*n* = 33; mPAP 35 mmHg). The profile of circulating metabolites among groups was compared using principal component analysis (PCA), analysis of covariance (ANCOVA), and Pearson’s correlation tests. Patients with noPH-HF and PH-HF were aged 64 ± 11 and 68 ± 10 years, respectively, with baseline left ventricular ejection fractions of 27 ± 7% and 26 ± 7%. Principal component analysis segregated groups, more markedly for PH-HF, with long-chain acylcarnitines, acetylcarnitine, and monounsaturated FA carrying the highest loading scores. After adjustment for age, sex, kidney function, insulin resistance, and N-terminal pro-brain natriuretic peptide (NT-proBNP), 5/15 and 8/15 lipid-related metabolite levels were significantly different from controls in noPH-HF and PH-HF subjects, respectively. All metabolites for which circulating levels interacted between group and NT-proBNP significantly correlated with NT-proBNP in HF-PH, but none with HF-noPH. FA-related metabolites were differently affected in HF with or without PH, and may convey adverse outcomes given their distinct correlation with NT-proBNP in the setting of PH.

## 1. Introduction

Heart failure (HF) carries a high societal burden, affecting 28 million individuals worldwide and is complicated, in approximately half of cases, by group 2 pulmonary hypertension (PH), which is a major determinant of prognosis [1,2]. To date, there exists no biomarker or pharmacological therapy specific for group 2 PH, hindering the capacity to predict and mitigate its complications such as right ventricular dysfunction, disabling symptoms, and death. While many group 2 PH cases solely reflect left heart pressure (isolated post-capillary PH), one third of patients develop a pre-capillary component due to pulmonary arterial vasoconstriction and alveolar-capillary remodeling (combined post- and pre-capillary PH). The process leading to vascular remodeling remains poorly understood, but is postulated to result from a combination of factors including the duration and severity of HF, genetic predisposition, environmental factors, and their associated metabolic perturbations.

The failing heart is described as an engine out of fuel, with impaired mitochondrial function, reduced levels of high-energy phosphate compounds, and metabolic remodeling characterized by decreased fatty acid (FA) oxidation and a relative increase in glucose metabolism [3]. Decreased cardiac FA uptake into the mitochondria, disturbed FA oxidation, and elevated circulating free FA have been associated with incident HF with reduced ejection fraction (HFrEF) and also correlate with the severity of HF [4,5]. Reflecting impaired mitochondrial FA utilization, numerous studies showed that circulating acylcarnitines, and long-chain acylcarnitines in particular, are increased in HFrEF and associated with adverse clinical outcomes [6,7,8].

Fatty acid metabolism is also disturbed in pulmonary arterial hypertension (PAH, group 1 PH), where an increase in circulating free FA may represent defective FA utilization and mitochondrial β-oxidation [9]. In terms of metabolomics data, findings resembling those seen in HF have been reported for group 1 PH; notably, an increase in circulating long-chain acylcarnitines [10,11]. Although there are limited metabolomics data available for group 2 PH, a study found long-chain acylcarnitines increased to a similar extent in PAH and group 2 PH [12]. However, whether the metabolic profile of HF patients diverge in those who develop PH, compared to those who remain with normal pulmonary pressure, remains unknown, and its association with disease severity has not been reported.

This study is a cross-sectional and sub-analysis of our previously published work [8,13]. We sought to characterize the profile of circulating energy-related metabolites in HF patients with (PH-HF) and without (noPH-HF) group 2 PH, compared to healthy individuals and evaluate whether a set of lipid-related metabolites are differentially regulated between both HF groups. In a cohort composed of 60 patients with HF and reduced ejection fraction (HFrEF) and 72 healthy subjects, we used a combination of targeted mass spectrometry-based methods and gas-chromatography with flame ionization detector (GC-FID) focusing on FA [13], organic acids, amino acids, and acylcarnitines [8].

## 2. Results

### 2.1. Population Characteristics

A total of 132 subjects were categorized as controls (*n* = 72), noPH-HF (*n* = 27, estimated mPAP = 21 ± 2 mmHg) and PH-HF (*n* = 33, estimated mPAP = 35 ± 6 mmHg). Baseline clinical characteristics for each group are reported in Table 1 and standard biochemical parameters in Table 2. Both HF groups displayed similar clinical features such as severely decreased left ventricular ejection fraction (LVEF) and high New York Heart Association (NYHA) class. However, patients with PH had higher NT-proBNP and uric acid levels, and more severely impaired kidney function reflected by higher plasma urea concentration and a trend towards lower estimated glomerular filtration rate (eGFR). Insulin resistance, estimated by homeostatic model assessment of insulin resistance (HOMA-IR), was more pronounced in noPH-HF. HF etiology did not differ among groups. NHYA classes were similarly distributed between HF groups and patients were adequately treated with neurohormonal blockade.

### 2.2. Differential Acylcarnitine and FA Profiles in noPH-HF and PH-HF Compared to Healthy Individuals

**Figure 1:**
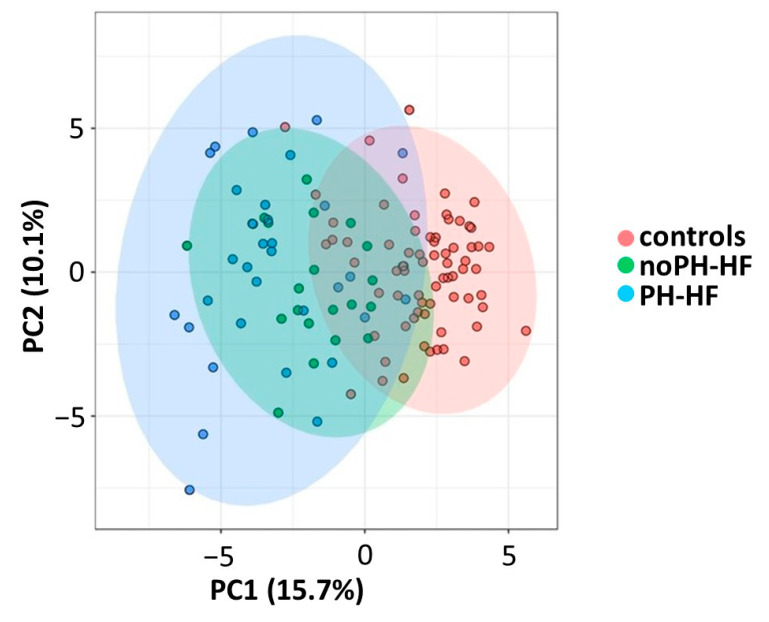
Principal component analysis identified distinct metabolic patterns in PH-HF (*n* = 33) and noPH-HF (*n* = 27) and segregated HF patients from healthy subjects (*n* = 72). A total of 55 variables were included in the analysis and comprised usual biochemical parameters as well as various metabolites measured by a combination of MS-based metabolomics approach targeting fatty acids, acylcarnitines, organic acids and amino acids. For the segregation between healthy subjects (controls) and HF patients, principal component 1 (PC1) and principal component 2 (PC2) accounted for 15.7% and 10.1% of the total variation, respectively. Controls are identified in red, noPH-HF in green and PH-HF in blue. Biplot analysis merging PCA plot and loadings plot identified the most potent metabolites in segregating controls (red) and noPH-HF (green) with PC1 and PC2 accounting for 11.9% and 9.3% respectively, and controls (red) and PH-HF (blue) with PC1 and PC2 accounting for 16.8% and 11.9% respectively. The corresponding loading scores in PC1 and PC2 for both analyses are reported in Table S1.

We first address the question of the specific metabolomic profile of each category of HF patients, either with or without PH, compared with healthy people. As reported in the PCA (Figure 1), patients with HF are segregated from controls (red) in the first principal component (PC1) which explained 15.7% of the variance. Specifically, noPH-HF patients (green) were mildly distanced from controls, and PH-HF patients (blue) spread further. The set of metabolites carrying most of the discriminant weight between controls vs noPH-HF (Table S1), and controls vs PH-HF (Table S1) was distinct for each comparison. Based on the top 15 metabolites with the higher absolute loading PC1, the majority of metabolites contributing to the separation between controls and noPH-HF were FA, particularly very long-chain FA (VLCFA: C20:0, C22:0 and C24 with a loading score from 0.19 to 0.27), monounsaturated FA (MUFA: C16:1Tn7 and C18:1n7 with a loading score of 0.17 and 0.18, respectively) and polyunsaturated FA (PUFA: C20:4n6, C18:2n6, C22:6n3 and C20:5n3 with a loading score between 0.17 and 0.28) (Supplemental Table S1). In contrast, although some of the latter FA were also involved in the differences between PH-HF and controls (C24:0, C22:0, C18:2n6 and C20:5n3), acylcarnitines more strongly distinguish PH-HF from controls. Specifically, long-chain acylcarnitines (LCACs: C16:0-AC, C18:1-AC, C18:2-AC), free carnitine (C0-AC) and C2-AC were among the metabolites underlying the highest magnitude of variance, discriminating between the groups on PC1 (loadings from 0.19 to 0.25). Commonly assessed and expected metabolites were also listed among discriminants in both HF groups, compared to controls, especially HDL- and LDL-cholesterol (Figure S1). From this unsupervised analysis, we observed that the specific metabolomic signature of each HF category, in this case according to the presence or absence of PH, differed, which led us to further analysis of the above-selected metabolites among groups.

### 2.3. Acylcarnitine and MUFA Perturbations Are Greater in PH-HF Compared to noPH-HF

**Figure 2:**
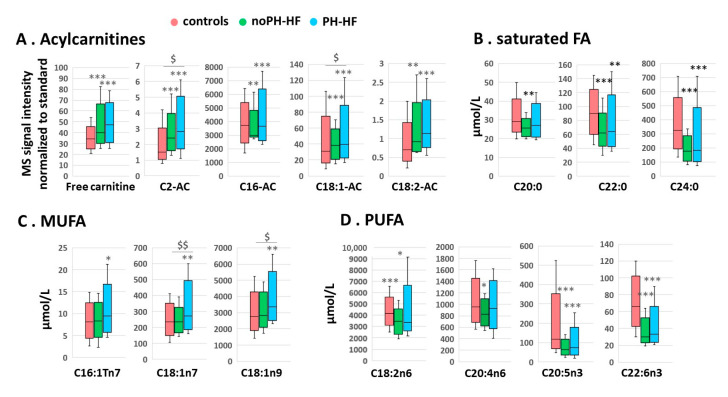
Acylcarnitines and fatty acids circulating levels are differentially affected in PH-HF (*n* = 33) and noPH-HF (N = 27) compared to controls (N = 72). Box plots represent top discriminant FA-related metabolites identified using the PCA analysis in the comparison noPH-HF (green) vs. controls (red) and PH-HF (blue) vs. controls. Shown are semi-quantitative analyses of (**A**) acylcarnitines reported as MS signal ratio normalized to standard, and the quantitative analysis of (**B**) saturated FA, (**C**) MUFA, and (**D**) PUFA. In the boxplots, rectangles represent the SD, the segment inside the rectangle the median and the whiskers above and below the maximum and minimum. * *p* < 0.05, ** *p* < 0.01, *** *p* < 0.001 compared to controls; ^$^ *p* < 0.05, ^$$^ *p* < 0.01 PH-HF vs. noPH-HF. Other more commonly measured metabolites are shown in Figure S1.

Based on the specific profile that emerged from the PCA analyses in each HF group, we conducted ANOVA analyses on selected acylcarnitines and FA. Long-chain acylcarnitines (C16-AC, C18:1-AC and C18:2-AC) were significantly increased by respectively 30%, 45%, 39% in noPH-HF and by 63%, 81%, 67% in PH-HF compared to controls (Figure 2A). Similarly, C0-AC and C2-AC were increased by 33% and 51% in noPH-HF, and by 43% and 99% in PH-HF. Moreover, C2-AC and C18:1-AC were significantly higher in PH-HF compared to noPH-HF. The FA profile, shown in Figure 2B–D, demonstrated saturated FA and PUFA levels similarly decreased in both HF groups compared to controls, apart from C20:0 and C20:4n6, which were lower only in noPH-HF by 14% and 13%, respectively. MUFA levels remained unchanged in the noPH-HF group, but a significant increase was observed in C16:1Tn7 (+20%), C18:1n7 (+26%) and C18:1n9 (+29%) in PH-HF.

We noticed that among the affected lipid-related metabolites, 4 were significantly more affected in HF-PH compared to HF-noPH: 2 acylcarnitines (C2-AC, C18:1AC) and 2 MUFA (C18:1n7 and C18:1n9). To further evaluate the differences between the HF groups, we conducted an additional PCA analysis comparing HF-noPH and HF-PH groups. We did not observe any major segregation in the first three PCs; however, some structure was observed between both HF groups on the fourth principal component (PC4) (Figure S2) that was still present after outlier removal. HF patients with PH (blue) appeared more heterogeneous than HF-noPH (green) on PC4, which accounts for 7.1% of the variance (Figure S3). The 4 metabolites with the highest absolute loading PC4 that drove the heterogeneous structure in the HF-PH group were the FA C18:1n7 as well as the acylcarnitine C18:1AC (Figure S4) already observed to be significantly different between HF groups (Figure 2), plus C22:5n3 and C22:4n6, which we added as metabolites of interest for our subsequent statistical analyses.

After adjustment for age, sex, eGFR and HOMA-IR, differences reported in Figure 2 remained significant for nearly all acylcarnitines and FA (Table 3, A. Analysis of Covariance ANCOVA 1). After adding NT-proBNP to the multivariate model, several differences in noPH-HF vs control groups were lost, while the significance of differences between PH-HF and controls in acylcarnitines and MUFA persisted (Table 3, ANCOVA 2).

### 2.4. Different Associations between Metabolites and NT-proBNP According to PH Status

To assess whether the relationship between metabolite concentration and NT-proBNP differed among groups, an interaction term between patient group and NT-proBNP, used as a surrogate for disease severity, was added to the ANCOVA 2 model (Table 4). Interactions were present for acylcarnitines, MUFA, and PUFA. After considering these interactions, the associations with NT-proBNP were significant only in the PH-HF group, irrespective of the metabolite considered: acylcarnitines (C2-AC, *p* < 0.01; C18:1-AC, *p* < 0.01; C18:2-AC, *p* < 0.05), MUFA (C18:1n7, *p* < 0.01; C18:1n9, *p* < 0.01), and PUFA (C20:4n6, *p* < 0.01; C20:5n3, *p* < 0.05). As these results may be affected by drug treatment, particularly those influencing fatty acid metabolism such as statins and diuretics, and the difficulty to have homogeneous groups in terms of treatment, we conducted additional interaction analyses and showed that none of the metabolites differentially regulated in both HF groups were affected by statin or diuretic treatment (Tables S2 and S3). As such a signature may also be closely related to the diabetic status, which is known to impact the FA and acylcarnitine profile, we conducted a last interaction analysis between patient group and the diabetic status. This interaction analysis did not showed any significant interaction effect (Table S4).

### 2.5. Metabolite Correlation with NT-proBNP Only in PH-HF

**Figure 3:**
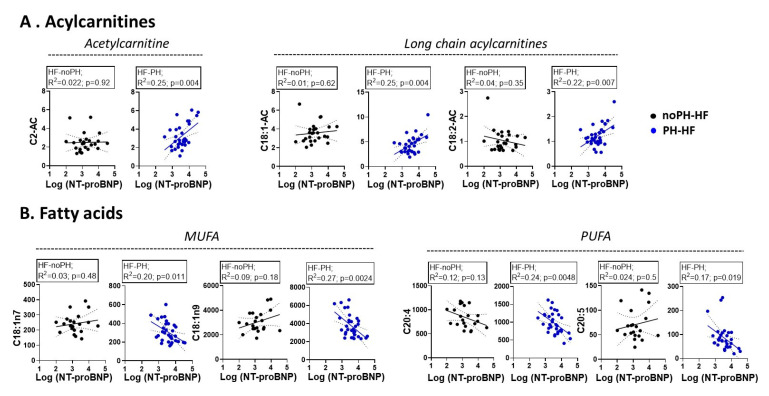
Acylcarnitines and fatty acids significantly correlate with NT-proBNP in PH-HF but not in noPH-HF patients. Pearson correlation analysis between NT-proBNP and (**A**) acylcarnitines, (**B**) FA (MUFA and PUFA) in noPH-HF (black) or PH-HF (blue) patients. The dotted lines indicate the 95% confidence intervals.

Correlation analyses were conducted to explore the relationship between disease severity (plasma NT-proBNP) and metabolites for which a significant interaction was found. Scatter plots for noPH-HF (black) and PH-HF (blue) are depicted in Figure 3. Similar to the interaction analyses, we observed no significant correlation between NT-proBNP and acylcarnitines or FA in noPH-HF patients. In contrast, there was a positive correlation between NT-proBNP and acylcarnitines in the PH-HF group, with C2-AC (R2 = 0.25, *p* = 0.004), C18:1AC (R2 = 0.25, *p* = 0.004), and C18:2AC (R2 = 0.22, *p* = 0.007). Regarding FA, negative correlations were observed in MUFA and PUFA as highlighted with C18:1n7 (R2 = 0.20, *p* = 0.011), C18:1n9 (R2 = 0.27, *p* = 0.024), C20:4 (R2 = 0.24, *p* = 0.0048), and C20:5 (R2 = 0.17, *p* = 0.019).

## 3. Discussion

This exploratory, cross-sectional study investigated circulating FA-related metabolites in HF patients with and without group 2 PH using a combination of semi-quantitative MS-based targeted approaches and GC-FID analysis. We found a clearly altered metabolic signature in HF patients, in which some metabolites were significantly more affected in the presence of PH, with regard to acylcarnitines and FA, that included the 2 acylcarnitines C2-AC and C18:1-AC and the 2 MUFA C18:1n7 and C18:1n9. Increased levels of acylcarnitines were found in both HF groups, albeit the magnitude of changes was more pronounced in the PH-HF group, specifically acetylcarnitine and long-chain acylcarnitines. With the exception of MUFA, which er lower only in PH-HF, FA were decreased in both HF groups. Adjustment for a marker of HF severity (plasma NT-proBNP) and factors known to affect lipid metabolism (age, sex, HOMA-IR, and eGFR) mitigated the difference in metabolite concentration between noPH-HF and controls; however, most differences between PH-HF and controls remained significant. These results suggest that this metabolic profile, indicative of disrupted FA metabolism, is more pronounced in group 2 PH compared to HF alone, and suggests that perturbations in FA metabolism could participate to the pathophysiological progression of HF towards HF with PH. This premise will require further exploration in the future. To our knowledge, this is the first study to describe the potential effect of group 2 PH on FA- and energy-related metabolites of HF patients, which furthers our understanding of individual variations in the development of PH. Except when appropriate, the discussion will not be restricted to the above-mentioned specific metabolites, but will include the broad family and particularly long chain acylcarnitines and MUFA.

Several studies have investigated the metabolome related to cellular energy production in PAH and HF, uncovering shared patterns of FA and acylcarnitine alterations across both diseases. Patients with HF exhibit long-chain acylcarnitine accumulation, which becomes more pronounced as LVEF declines, thereby showing an association between marked metabolic perturbations and advanced HF [14]. Previous analyses from the current patient cohort demonstrated increased acylcarnitines in HFrEF, including the very-long-chain acylcarnitine C26:0 and dicarboxylic acylcarnitines, revealing that defects in FA oxidation are affected beyond the mitochondria and involve other cellular compartments such as peroxisomes [8]. In addition, a global change in circulating free FA levels was demonstrated in HF [4], including increased MUFA and saturated FA, as well as decreased PUFA levels [5,13].

In PAH, in addition to elevated circulating free FA, increased long-chain acylcarnitines, including the C18:1-AC, were reported in multiple studies [10,15]. Highlighting their importance, one comprehensive metabolomic analysis showed that acylcarnitines, including the C18:1-AC as we reported here, represented 3/16 (19%) of discriminating and prognostic metabolites between PAH and controls [11]. It is postulated that the excess of acylcarnitines results from overstimulation of the mitochondrial fatty acid beta-oxidation pathway to comply with increasing right ventricular demand. Another hypothesis implies a defective mitochondrial pathway leading to failure of FA utilization and subsequent acylcarnitine accumulation, and is supported by a decreased expression of genes encoding for enzymes involved in fatty acid β-oxidation in right ventricular tissue in the presence of PH [16].

In group 2 PH, FA metabolism has been incompletely investigated. In a single study exploring the metabolome of group 2 PH, a similar degree of long-chain acylcarnitines increase in both PAH and group 2 PH was observed [12]. This suggests shared mitochondrial FA metabolism dysfunction across these two PH groups, yet these findings were not compared to HF without PH. In the present study, we found that HF with and without PH both exhibited defects on FA metabolism, but with distinct lipid profiles.

Following the identification of discriminating FA and acylcarnitines, we examined whether the correlation between metabolite and NT-proBNP was influenced by patient group. We found that when an interaction between metabolite and HF group was present, the correlation was present only present in PH-HF. In addition to its association with left-sided HF, NT-proBNP also reflects right ventricular strain and has independent prognostic value in PH [17]. Therefore, in group 2 PH, MUFA, particularly C18:1n7 and C18:1n9, and long chain acylcarnitines, mostly the C18:1-AC, could reflect or contribute to complications in a way that does not occur in HF when pulmonary hypertension is not established. Of interest in light of our results, high levels of MUFA such as C18:1n7 and, to a lesser extent, C18:1n9 have been significantly associated with total mortality in a cohort of 183 patients with stable symptomatic HF [5]. Similar observations were made regarding long chain acylcarnitines which are associated to adverse clinical outcomes in chronic HF [6] and a predictor of cardiovascular mortality in incident dialysis patients [7]. Additionally, increased levels of long chain acylcarnitines, C18:1-AC in particular, returned to normal following mechanical circulatory support [6].

Our findings are in agreement with other groups that have associated medium- and long-chain acylcarnitines with adverse outcomes across a variety of cardiovascular diseases, including HF and PH [6,18]. Long-chain acylcarnitines may impact clinical outcomes through diverse pathways. In myocytes, they increase intracellular calcium through activation of ion channels in the sarcoplasmic reticulum [19], causing ventricular arrhythmogenicity, as was demonstrated in children with inborn errors of FA oxidation [20]. Interestingly, changes in calcium influx could also be involved in the regulation of arterial smooth muscle cell contraction and increase vascular resistance, a pathologic hallmark of group 2 PH. Indeed, Criddle et al. showed that palmitoylcarnitine, a long-chain acylcarnitine, induces coronary artery vasoconstriction in isolated rat hearts [21,22]. Such properties have not been studied in lung vessels, though similar vasodynamic effects in the alveolar-capillary bed could potentially modulate pulmonary artery pressures in group 2 PH. Finally, long-chain acylcarnitines have previously been associated with hyperuricemia, an independent marker of poor prognosis in HF. Although uric acid did not correlate with acylcarnitine concentration (data not shown), hyperuricemia was more prevalent in the HF-PH group [23].

On the other end, FA depletion is thought to depress cardiac work through a lack of energetic substrate [24]. Nevertheless, benefits of PUFA in vascular physiology, particularly n3-PUFA, have been documented and shown to exert vasoprotective effects as well as to limit abnormal vascular growth [25,26]. In this study, although speculative, the decrease of n-3 PUFA may, in contrast, exacerbate vascular resistance and contribute to PH. Moreover, a recent study in isolated mesenteric and femoral arteries showed that while saturated FA increased α-adrenergic contraction of systemic arteries, PUFA resulted in the opposite effect [27]. However, to the best of our knowledge, the effect of acute and long-term exposure to n-7 and n-9 MUFA, on vascular function has not been investigated. Clearly, the differential impact of saturated FA, MUFA, and PUFA on vascular function merits further exploration.

Clinically, our exploratory and cross-sectional cohort exemplifies the group 2 PH conundrum, where despite optimization of neurohormonal blockade, effective therapies targeted to PH are lacking. Severe PH may preclude candidacy to advanced heart failure therapies such as cardiac transplantation, highlighting the importance of developing valid tools to predict its emergence. Specific defects in FA utilization, recognized by changes in FA and acylcarnitines, in combination or in addition to NT-proBNP, may help identify patients before onset or at an early stage of PH. Since therapies aimed at group 2 PH have proven largely unsuccessful in mitigating cardiovascular outcomes, characterizing changes to the metabolome may help identify patients who would benefit from such therapy, or contribute to drug development aimed at PH etiology, rather than palliation with vasodilators.

### Study Limitations

We acknoledge the exploratory and observational nature of the study. The primary goal of this study was to evaluate the relevance of further exploring the role of disturbed FA metabolism in the development of pulmonary hypertension in heart failure. In line with this limitation, we recognize that the small number of subjects limit the power to identify significant differences. In addition, because of the cross-sectional design, we were unable to perform outcomes analysis of the clinical evolution of HF, nor could we determine the existence of a causal relationship between the identified metabolites and the development of PH.

The combination of methodological approaches used in this analysis did not allow for an exhaustive coverage of lipids, for which there may exist significant but unmeasured associations. Although we adjusted for variables known to affect the circulating metabolite levels, the observational nature and limited sample size of this study cannot fully account for confounding factors. Our HF groups did not differ significantly in terms of LVEF, NYHA class, or pharmacological treatment. However, a trend towards lower eGFR in the PH-HF group may have influenced the relationship between metabolites and NT-proBNP, as the latter accumulates in kidney disease. To that effect, a correlation analysis between metabolite concentration and eGFR (Figure S2) yielded no significant findings, decreasing the likelihood that differences in kidney function affected or findings.

As the present study was restricted to subjects with HFrEF, our findings may not apply to group 2 PH subjects with preserved left ventricular ejection fraction (HFpEF) or primary valvular heart disease. Finally, diagnosis of PH was ascertained retrospectively using echocardiography, providing a reliable estimation of systolic pulmonary artery pressure, however without matching the precision achieved with right heart catheterization, nor providing information on pulmonary vascular resistance. Misclassification of subjects with PH may have occurred in patients with an incomplete tricuspid regurgitation (TR) signal, although the magnitude of this bias is thought to be low and non-differential.

## 4. Materials and Methods

### 4.1. Participants and Sample Collection

This was a cross-sectional and exploratory analysis from a cohort designed to investigate the circulating metabolome of HFrEF patients, from which detailed inclusion and exclusion criteria have been previously published [8,13]. Briefly, patients screened at the HF clinic of the Montreal Heart Institute were included if they were aged ≥ 45 years and diagnosed with HFrEF based on chronic HF symptoms and LVEF ≤ 40%. Patients who underwent heart surgery in the 3 months preceding blood sampling and those without an echocardiogram within 2 years of study participation were excluded. The control cohort was composed of healthy individuals aged ≥ 45 years without established cardiovascular disease or risk factors for such including a body mass index ≥ 32 kg/m^2^, diabetes, smoking, untreated hypertension or untreated hyperlipidemia. Recruitment of the control group occurred in the catheterization laboratory among patients who presented with non-specific cardiac symptoms, after having ruled out cardiovascular disease.

As per study protocol, each subject fasted for 12 h before undergoing a 20 mL peripheral venous blood draw on ice. Blood was centrifuged, frozen in separate aliquots, and stored at −80°C. Aliquots were only thawed for analysis: no refrozen aliquots were analyzed. Each patient gave written informed consent and the study was approved by the Montreal Heart Institute Institutional Review Board.

### 4.2. Data Collection and Patient Classification

Clinical, echocardiographic, and pharmacological data was collected at the time of blood sampling after clinical evaluation by physician scientists. The echocardiogram performed closest to study enrollment was reviewed for systolic pulmonary artery pressure (sPAP) and left ventricular ejection fraction (LVEF). sPAP was estimated using the tricuspid valve peak systolic pressure gradient added to mean right atrial pressure, as per the American Society of Echocardiography guidelines [28]. While the gold standard diagnosis for PH involves right heart catheterization showing a mean pulmonary artery pressure (mPAP) > 20 mmHg [29], mean pressure was inferred using echocardiography sPAP with a validated formula [mPAP = 0.61 × sPAP + 2] [30,31]. In this study, PH was defined as sPAP ≥ 40 mmHg on echocardiography, corresponding to an estimated mPAP ≥ 26mmHg. Patients in whom the absence of tricuspid regurgitation precluded estimation of pulmonary pressure were categorized as sPAP < 40 mmHg, an assumption based on data showing that patients with established PH develop sufficient tricuspid regurgitation to measure sPAP in 94% cases [32]. The cutoff of 40 mmHg for sPAP was chosen to reflect the prevailing diagnosis guidelines for PH at the time of study design and approval (mPAP > 25 mmHg). All patients with PH were assumed to have group 2 PH given reduced LVEF. Patients were classified into 3 groups: (1) noPH-HF (LVEF ≤ 40% and sPAP < 40 mmHg), (2) PH-HF (LVEF ≤ 40% and sPAP > 40 mmHg), and (3) controls (presumed normal LVEF and pulmonary artery pressure) (Table 1). In addition, patients without an echocardiogram within 2 years of study participation (plus or minus one year) were not included. Eight patients were then excluded because of this criterion, explaining the lower number of HF patients (*n* = 60) compared to our previous studies (*n* = 68). The number of healthy subjects remains the same (*n* = 72).

### 4.3. Metabolite Profiling

Data for all plasma metabolites included in this study were assessed by various methods as reported in previous publications and include the following: (i) commonly assessed metabolites, including those related to cholesterol, triglycerides, glucose, insulin and glycerol, using commercial biochemical assays [8,13] (Table 2); (ii) 29 FA reflecting the total pool of FA (free + bound FA), by gas chromatography (GC) combined with flame ionization detection [13], (iii) 8 organic acids (ketone bodies, lactate, pyruvate and Krebs cycle intermediates) and 12 amino acids using targeted quantitative GC coupled to mass spectrometry (MS) [8], and 14 acylcarnitines, which are proxies of altered FA utilization and oxidation, using shotgun MS/MS [8]. The resulting database comprised 71 metabolites, which were used to phenotype the HF subgroups. Missing more than 25% of the values were removed, and metabolites with more than 10% missing values were excluded. The remaining missing values were imputed using the K-Nearest Neighbors approach using MetaboAnalystR 3.0 (www.metaboanalyst.ca, accessed on 23 March 2021) and non-informative (redundant) metabolites were filtered out using interquartile range [33]. After these pre-processing steps, we ended up with 55 metabolites in 124 samples. Raw concentrations were subjected to auto-scaling and were log transformed, to obtain normalized metabolite concentrations or ratio.

### 4.4. Statistical Analyses

Population characteristics and laboratory parameters were presented according to control, noPH-HF, and PH-HF groups. Continuous variables were presented as means ± standard deviation or medians and interquartile range according to distribution normality. Categorical variables were presented as frequencies and percentages. Groups were compared using analysis of variance, Kruskal Wallis, Chi-squared, or Fisher’s exact tests where appropriate (Graph Pad Prism 8.3.1 software). Principal component analysis (PCA) was performed on normalized metabolite concentrations for the 55 metabolites and 124 samples remaining post-quality control, to visually assess how the different populations were discriminated using the prcomp function and the ggbiplot package in R (version 3.4.4, https://github.com/vqv/ggbiplot, accessed on 23 March 2021). To identify metabolites with the ability to discriminate subjects among groups, loadings of PC1 and PC2 were used to select metabolites with the highest PC and their mean plasma concentration or ratio was compared among groups using analysis of variance (ANOVA). They were then compared using stepwise analysis of covariance (ANCOVA) multivariable models considering preselected variables known to affect circulating levels of lipid metabolites [34,35]. The first model (ANCOVA 1) included age, sex, estimated glomerular filtration rate (eGFR) and homeostatic model assessment of insulin resistance (HOMA-IR) as covariates. The second model (ANCOVA 2) included the same covariates in addition to NT-proBNP, used as a surrogate for HF severity. The interaction between patient group and NT-proBNP was tested to explore the effect of patient group on the association between NT-proBNP and metabolites. Interaction analyses were also tested with diuretics, statins, and diabetic status. Metabolites with significant interaction effects were further investigated with Pearson correlation tests for their association with NT-proBNP in each patient group. Normality and linearity were verified, and logarithmic transformation was applied where indicated. Two-tailed *p*-values < 0.05 were considered statistically significant and there was no correction or adjustment for multiple testing made. Data analyses were performed using SAS software (version 9.4, SAS Institute, Cary, NC, USA).

## 5. Conclusions

The metabolic signature of HFrEF with group 2 PH indicates a disruption of FA metabolism that is more pronounced than in HFrEF without PH, with differences in some circulating long-chain acylcarnitines, acetylcarnitine, and MUFA. These metabolites correlate with NT-proBNP only in PH-HF subjects, which supports their potential implication in the development and progression of PH. The role of defects in FA metabolism in vascular remodeling and vasoreactivity merits further investigation. To that end, future studies focusing on HF and PH may benefit from pulmonary vascular resistance measures, as well as pre- and post-lung circulation blood samples. This cross-sectional and observational study highlighted the importance of subcategorizing HF patients to better understand the course of HF and PH and opens up important research avenues to be explored in the future.

## Figures and Tables

**Table 1:** Baseline characteristics by heart failure and pulmonary hypertension status.

	Control	noPH-HF	PH-HF	*p*-Value ^a^	*p*-Value ^b^
Characteristics	(*n* = 72)	(*n* = 27)	(*n* = 33)		
Age, years	59 ± 9	64 ± 11	68 ± 10	NS	<0.01
Female sex, *n* (%)	37 (51)	18 (67)	26 (79)	NS	0.02
Body mass index, kg/m2	26 ± 3	27 ± 4	27 ± 4	NS	0.50
Ischemic cardiomyopathy, *n* (%)	0 (0)	14 (52)	23 (70)	NS	<0.01
Type 2 diabetes, *n* (%)	0 (0)	11 (41)	17 (52)	NS	0.01
Smoking, *n* (%)	0 (0)	3 (11)	5 (15)	NS	<0.01
NYHA class 1, *n* (%)	NA	1 (4)	1 (4)	NS	NA
NYHA class 2, *n* (%)	NA	12 (44)	19 (57)	NS	NA
NYHA class 3, *n* (%)	NA	14 (52)	13 (39)	NS	NA
Systolic blood pressure, mmHg	120 ± 13	107 ± 17	106 ± 18	NS	<0.01
Diastolic blood pressure, mmHg	73 ± 8	60 ± 6	56 ± 9	0.051	<0.01
Echocardiographic parameters					
LVEF, %	NA	27 ± 7	26 ± 7	NS	NA
RV dysfunction, *n* (%)	NA	2 (7)	7 (21)	NS	NA
sPAP, mmHg	NA	31 ± 3	54 ± 10	<0.01	NA
mPAP, mmHg	NA	21 ± 2	35 ± 6	<0.01	NA
Medications					
Beta-blockers, *n* (%)	3 (4)	22 (81)	27 (82)	NS	<0.01
ACE inhibitor, *n* (%)	7 (10)	21 (78)	29 (88)	NS	<0.01
Mineralocorticoid receptor antagonist, *n* (%)	0 (0)	16 (59)	23 (70)	NS	<0.01
Diuretics, *n* (%)	5 (7)	24 (89)	32 (97)	NS	<0.01
Digoxin, *n* (%)	0 (0)	16 (59)	22 (67)	NS	<0.01
Nitrate, *n* (%)	0 (0)	16 (59)	14 (42)	NS	<0.01
Amiodarone, *n* (%)	0 (0)	9 (33)	9 (27)	NS	<0.01
Calcium channel blocker, *n* (%)	3 (4)	1 (4)	2 (6)	NS	NS
Aspirin, *n* (%)	10 (14)	12 (44)	20 (61)	NS	<0.01
Warfarin, *n* (%)	1 (1)	11 (41)	17 (52)	NS	<0.01
Statin, *n* (%)	7 (24)	19 (70)	25 (76)	NS	<0.01
Oral hypoglycemic agent, *n* (%)	0 (0)	8 (30)	13 (39)	NS	<0.01
Levothyroxin, *n* (%)	6 (8)	7 (26)	6 (18)	NS	0.07
Allopurinol, *n* (%)	1 (1)	6 (22)	11 (33)	NS	<0.01

^a^ refers to *p* values comparing noPH-HF and PH-HF; ^b^ refers to *p* values comparing all three groups. Diuretics included furosemide and thiazides; lipid-lowering agents included statins and fibrates. Groups were compared using ANOVA, Kruskal Wallis, Chi-square or Fisher’s exact test where appropriate. HF-noPH denotes heart failure without pulmonary hypertension; HF-PH, heart failure with pulmonary hypertension; NYHA, New York Heart Association; LVEF, left ventricular ejection fraction; sPAP, systolic pulmonary artery pressure; mPAP, mean pulmonary artery pressure; ACE, angiotensin-converting enzyme; NA, not applicable.

**Table 2:** Standard biochemical characteristics at baseline by heart failure and pulmonary hypertension status.

	Control (*n* = 72)	noPH-HF (*n* = 27)	PH-HF (*n* = 33)	*p*-Value ^a^	*p*-Value ^b^
Biochemical Parameters					
Hemoglobin, g/L	142 ± 10	132 ± 15	134 ± 15	NS	<0.01
Leucocytes, count × 10^9^/L	5.8 ± 1.3	7.6 ± 2.0	7.4 ± 1.8	NS	<0.01
Urea, nM	6.1 ± 1.4	10.1 ± 3.7	12.8 ± 4.9	<0.01	<0.01
Estimated glomerular filtration rate, mL/min	83 ± 21	55 ± 25	46 ± 20	NS	<0.01
NT-proBNP, ng/mL	57 ± (30–90)	1273 (733–3377)	3704 (1877–5954)	<0.01	<0.01
Elevated troponin, *n* (%)	0 (0)	4 (15)	6 (18)	NS	<0.01
Asparagine aminotransferase, U/L	21 ± 6	24 ± 12	24 ± 8	NS	NS
Alanine aminotransferase, U/L	40 ± 9	42 ± 16	39 ± 11	NS	NS
Alkaline phosphatase, U/L	71 ± 18	112 ± 53	99 ± 42	NS	<0.01
Total bilirubin, μM	11 ± 5	12 ± 6	13 ± 9	NS	NS
Uric acid, μM	281 ± 77	394 ± 127	468 ± 136	0.04	<0.01
Total cholesterol, mM	5.1 ± 0.8	4.4 ± 0.9	3.9 ± 1.2	NS	<0.01
HDL-cholesterol, mM	1.5 ± 0.4	1.0 ± 0.3	0.9 ± 0.2	NS	<0.01
LDL-cholesterol, mM	3.2 ± 0.7	2.6 ± 0.7	2.4 ± 1.1	NS	<0.01
Triglycerides, mM	1.1 ± 0.5	2.0 ± 1.3	1.4 ± 0.7	NS	<0.01
Glucose, mM	4.6 ± 0.5	7.4 ± 3.1	7.0 ± 1.9	NS	<0.01
HOMA-IR	23 ± 12	65 ± 40	45 ± 26	0.03	<0.01
C-reactive protein, μg/mL	1.0 (0.5–2.1)	2.9 (1.2–11.3)	3.4 (2.0–9.3)	NS	<0.01
TNF-α, pg/mL	1.1 ± 0.4	2.1 ± 1.0	2.2 ± 0.6	NS	<0.01
Myeloperoxydase, ng/mL	16.9 ± 6.7	23.1 ± 8.5	23.2 ± 15.0	NS	<0.01

^a^ refers to *p* values comparing noPH-HF and PH-HF; ^b^ refers to *p* values comparing all three groups. Groups were compared using ANOVA, Kruskal Wallis, Chi-square or Fisher’s exact test where appropriate.

**Table 3:** Adjusted comparison of circulating acylcarnitine and fatty acid levels among groups.

**A. ANCOVA 1 (Sex, Age, HOMA-IR, eGFR)**			
	**noPH-HF vs. Controls**	**PH-HF vs. Controls**	**PH-HF vs. noPH-HF**
Acylcarnitines			
Free carnitine	<0.01	<0.001	NS
*C2-AC*	<0.01	<0.001	<0.05
*C16-AC*	<0.05	<0.01	NS
*C18:1-AC*	<0.01	<0.001	0.07
*C18:2-AC*	<0.01	<0.001	NS
Saturated fatty acids			
*C20:0*	NS	NS	<0.05
*C22:0*	<0.01	<0.05	NS
*C24:0*	<0.001	<0.001	NS
Monounsaturated fatty acids			
*C16:1Tn7*	NS	NS	NS
*C18:1n7*	NS	<0.05	<0.01
*C18:1n9*	NS	<0.05	<0.01
Polyunsaturated fatty acids			
*C18:2n6*	<0.05	NS	NS
*C20:4n6*	NS	NS	<0.05
*C20:5n3*	<0.001	<0.01	NS
*C22:5n3*	<0.05	NS	<0.05
*C22:4n6*	NS	<0.05	<0.05
*C22:6n3*	<0.001	<0.05	NS
**B. ANCOVA 2 (Sex, Age, HOMA-IR, eGFR, NT-proBNP)**			
	**noPH-HF vs. Controls**	**PH-HF vs. Controls**	**PH-HF vs. noPH-HF**
Acylcarnitines			
*Free carnitine*	<0.05	<0.01	NS
*C2-AC*	NS	<0.01	0.06
*C16-AC*	NS	<0.05	NS
*C18:1-AC*	<0.01	<0.001	0.07
*C18:2-AC*	<0.05	<0.001	NS
Saturated fatty acids			
*C20:0*	NS	NS	<0.05
*C22:0*	NS	NS	NS
*C24:0*	<0.001	<0.001	NS
Monounsaturated fatty acids			
*C16:1Tn7*	NS	<0.05	NS
*C18:1n7*	NS	<0.05	<0.01
*C18:1n9*	NS	<0.01	<0.01
Polyunsaturated fatty acids			
*C18:2n6*	NS	NS	NS
*C20:4n6*	NS	NS	<0.05
*C20:5n3*	<0.01	NS	NS
*C22:5n3*	NS	NS	NS
*C22:4n6*	NS	NS	NS
*C22:6n3*	*<0.05*	*NS*	*<0.05*

Pre-specified variables included the ANCOVA 1 model were age, sex, eGFR, HOMA-IR; ANCOVA 2 adjusted for age, sex, eGFR, HOMA-IR and NT-proBNP. NT-proBNP was log-transformed. ANCOVA denotes analysis of covariance; eGFR, estimated glomerular filtration rate; HOMA-IR, homeostatic model assessment of insulin resistance; NS, non significant. *p*-values are derived from the ANCOVA model and refer to the comparison of plasma metabolites among groups.

**Table 4:** Interaction analysis with NT-proBNP.

Interaction with NT-proBNP		
	noPH-HF	PH-HF
Acylcarnitines		
free carnitine	NS	NS
C2-AC	NS	<0.01
C16-AC	NS	NS
C18:1-AC	NS	<0.01
C18:2-AC	NS	<0.05
Saturated fatty acids		
C20:0	NS	NS
C22:0	NS	NS
C24:0	NS	NS
Monounsaturated fatty acids		
C16:1Tn7	NS	NS
C18:1n7	NS	<0.01
C18:1n9	NS	<0.01
Polyunsaturated fatty acids		
C18:2n6	NS	NS
C20:4n6	NS	<0.01
C20:5n3	NS	<0.05
C22:4n6	NS	NS
C22:5n3	NS	NS
C22:6n3	NS	NS

When a significant interaction was identified, the correlation between metabolite and NT-proBNP was tested in in both noPH-HF and PF-HF groups. The corresponding *p* values are shown.

## Data Availability

The data presented in this study are available on request from the corresponding author. The data are not publicly available due to privacy and ethical.

## Supplementary Materials

The following are available online at https://www.mdpi.com/article/10.3390/metabo11040196/s1, Figure S1: Other metabolites that are differentially changed in PH-HF (*n* = 33) and noPH-HF (*n* = 27) compared to controls (*n* = 72), Figure S2: PCA analysis reporting all the PC combination, Figure S3: Principal component analysis identified a higher heterogeneity in PH-HF (*n* = 33) compared to noPH-HF (*n* = 27), Figure S4: Loading plot analysis showing the metabolites used for the PCA analysis, Figure S5: Acylcarnitines and fatty acids do not correlate with eGFR in PH-HF and noPH-HF, Table S1: Loading scores from the PCA analysis for the comparison noPH-HF vs. controls and PH-HF vs. controls, Table S2. Interaction analysis with statins treatment, Table S3. Interaction analysis with diuretics treatment, Table S4. Interaction analysis with diabetic (type 2 diabetes) status.
