# Supplementary material for: Long-Chain Acylcarnitines and Monounsaturated Fatty Acids Discriminate Heart Failure Patients According to Pulmonary Hypertension Status
Source: Metabolites. 2021 Mar 26;11(4):196. doi: 10.3390/metabo11040196 (PMC8066759; doi:10.3390/metabo11040196)
Supplement: Supplementary file 1 [file metabolites-11-00196-s001.pdf]

*Article*

# Long-Chain Acylcarnitines and Monounsaturated Fatty Acids Discriminate Heart Failure Patients According to Pulmonary Hypertension Status

**SUPPLEMENTARY MATERIAL**

**Table S1. Loading scores from the PCA analysis for the comparison noPH-HF vs controls and PH-HF vs controls.**
*A. Comparison noPH-HF and controls*

| Metabolites | PC1 loading score | PC2 loading score |
|---|---|---|
| *citrate* | -0.198 | -0.192 |
| *glucose* | -0.192 | -0.166 |
| *C18:1n7_FA* | -0.184 | 0.027 |
| *C0_car* | -0.175 | -0.075 |
| *TG* | -0.163 | 0.181 |
| *pyruvate* | -0.153 | -0.189 |
| *glycerol* | -0.144 | -0.120 |
| *C18:1n9_FA* | -0.133 | 0.146 |
| *C18:1_car* | -0.127 | -0.135 |
| *C16:1n7_FA* | -0.126 | 0.207 |
| *C3_car* | -0.123 | -0.036 |
| *C16_car* | -0.121 | -0.076 |
| *lactate* | -0.120 | -0.258 |
| *C18_car* | -0.108 | -0.079 |
| *succinate* | -0.107 | -0.110 |
| *C22:1n9_FA* | -0.104 | -0.081 |
| *C2_car* | -0.099 | -0.094 |
| *C18:2_car* | -0.091 | -0.084 |
| *C20:3n6_FA* | -0.075 | 0.122 |
| *C20:3n9_FA* | -0.073 | 0.133 |
| *C18:1n9T_FA* | -0.069 | 0.043 |
| *C22:4n6_FA* | -0.066 | 0.165 |
| *insulin* | -0.063 | -0.236 |
| *C18:0_FA* | -0.061 | 0.040 |
| *C16:0_FA* | -0.058 | 0.179 |

| | | |
|---|---|---|
| *C18:3n6G_FA* | -0.057 | 0.265 |
| *C14:0_FA* | -0.056 | 0.326 |
| *C5_car* | -0.040 | -0.064 |
| *C12:0_FA* | -0.032 | 0.118 |
| *C18:3n3a_FA* | -0.024 | 0.152 |
| *C10:0_FA* | -0.021 | 0.067 |
| *C14_car* | -0.021 | 0.002 |
| *AcAc* | 0.004 | -0.129 |
| *fumarate* | 0.009 | -0.022 |
| *B-HB* | 0.017 | -0.101 |
| *C20:1n9_FA* | 0.031 | 0.133 |
| *C4_car* | 0.045 | -0.017 |
| *FFA* | 0.048 | 0.081 |
| *C22:5n3_FA* | 0.056 | 0.170 |
| *C22:2n6_FA* | 0.060 | 0.019 |
| *C20:3n3_FA* | 0.065 | 0.250 |
| *C10_car* | 0.071 | -0.077 |
| *C24:1n9_FA* | 0.113 | -0.193 |
| *C20:2n6_FA* | 0.117 | 0.048 |
| *total-cholesterol* | 0.159 | -0.042 |
| *C20:4n6_FA* | 0.166 | 0.084 |
| *C16:1n7T_FA* | 0.173 | 0.072 |
| *C20:0_FA* | 0.191 | -0.158 |
| *LDL-cholesterol* | 0.198 | -0.016 |
| *C18:2n6_FA* | 0.205 | -0.185 |
| *C22:0_FA* | 0.219 | -0.055 |
| *C24:0_FA* | 0.269 | -0.030 |
| *HDL-cholesterol* | 0.270 | 0.014 |
| *C22:6n3_FA* | 0.281 | -0.098 |
| *C20:5n3_FA* | 0.283 | 0.000 |

*B. Comparison PH-HF and controls*

| **Metabolites** | **PC1 loading score** | **PC2 loading score** |
|---|---|---|
| *C18:1_car* | -0.245 | -0.141 |

| | | |
|---|---|---|
| ***citrate*** | -0.214 | -0.166 |
| ***C2_car*** | -0.204 | -0.074 |
| ***C16_car*** | -0.192 | -0.090 |
| ***C0_car*** | -0.189 | -0.030 |
| ***C18:2_car*** | -0.186 | -0.043 |
| ***C18:1n9_FA*** | -0.166 | 0.103 |
| ***C22:4n6_FA*** | -0.148 | 0.171 |
| ***C18:1n7_FA*** | -0.145 | 0.037 |
| ***C18_car*** | -0.143 | -0.076 |
| ***glucose*** | -0.139 | -0.144 |
| ***fumarate*** | -0.078 | -0.115 |
| ***C16:1n7_FA*** | -0.077 | 0.212 |
| ***C3_car*** | -0.074 | 0.011 |
| ***C14_car*** | -0.068 | 0.026 |
| ***B-HB*** | -0.067 | -0.246 |
| ***pyruvate*** | -0.065 | -0.055 |
| ***C18:1n9T_FA*** | -0.065 | -0.006 |
| ***C20:3n9_FA*** | -0.064 | 0.271 |
| ***C16:0_FA*** | -0.058 | 0.178 |
| ***lactate*** | -0.056 | -0.122 |
| ***AcAc*** | -0.054 | -0.250 |
| ***C12:0_FA*** | -0.046 | 0.094 |
| ***glycerol*** | -0.045 | -0.111 |
| ***C14:0_FA*** | -0.032 | 0.293 |
| ***C22:1n9_FA*** | -0.030 | 0.072 |
| ***TG*** | -0.026 | 0.160 |
| ***C18:0_FA*** | -0.026 | -0.009 |
| ***succinate*** | -0.025 | -0.134 |
| ***C10:0_FA*** | -0.022 | 0.048 |
| ***C20:1n9_FA*** | -0.021 | 0.112 |
| ***C18:3n6G_FA*** | -0.009 | 0.309 |
| ***C22:2n6_FA*** | -0.002 | -0.014 |
| ***C5_car*** | 0.021 | 0.105 |
| ***C4_car*** | 0.031 | 0.034 |
| ***C20:3n6_FA*** | 0.038 | 0.161 |

| | | |
|---|---|---|
| *insulin* | 0.039 | -0.004 |
| *C18:3n3a_FA* | 0.055 | 0.095 |
| *C10_car* | 0.059 | -0.062 |
| *C22:5n3_FA* | 0.068 | 0.244 |
| *C16:1n7T_FA* | 0.072 | -0.037 |
| *C20:4n6_FA* | 0.080 | 0.047 |
| *C24:1n9_FA* | 0.081 | -0.210 |
| *FFA* | 0.091 | -0.067 |
| *C20:3n3_FA* | 0.145 | 0.182 |
| *C20:0_FA* | 0.159 | -0.162 |
| *C20:2n6_FA* | 0.163 | -0.031 |
| *C18:2n6_FA* | 0.191 | -0.239 |
| *C22:0_FA* | 0.199 | -0.054 |
| *C20:5n3_FA* | 0.224 | 0.070 |
| *C22:6n3_FA* | 0.230 | -0.050 |
| *C24:0_FA* | 0.245 | -0.069 |
| *LDL-cholesterol* | 0.265 | -0.036 |
| *HDL-cholesterol* | 0.270 | 0.009 |
| *total-cholesterol* | 0.284 | -0.035 |

In grey, the top 15 metabolites with the higher relative score plot

**Table S2. Interaction analysis with statins treatment**

| | ***Statins*** | |
|---|---|---|
| | *noPH-HF* | *PH-HF* |
| | **Acylcarnitines** | |
| **free carnitine** | NS | NS |
| **C2-AC** | NS | NS |
| **C16-AC** | NS | NS |
| **C18:1-AC** | NS | NS |
| **C18:2-AC** | NS | NS |
| | **Saturated fatty acids** | |
| **C20:0** | NS | NS |

| | | |
|---|---|---|
| **C22:0** | NS | NS |
| **C24:0** | NS | NS |
| **Monounsaturated fatty acids** | | |
| **C16:1Tn7** | NS | NS |
| **C18:1n7** | NS | NS |
| **C18:1n9** | NS | NS |
| **Polyunsaturated fatty acids** | | |
| **C18:2n6** | NS | NS |
| **C20:4n6** | NS | NS |
| **C20:5n3** | NS | NS |
| **C22:4n6** | NS | NS |
| **C22:5n3** | NS | NS |
| **C22:6n3** | NS | NS |

In the presence of significant interaction, the correlation between metabolite and NT-proBNP was tested in each group and the corresponding *P* values were noted. NS: not significant.

**Table S3. Interaction analysis with diuretics treatment**

| | ***Diuretics*** | |
|---|---|---|
| | noPH-HF | PH-HF |
| **Acylcarnitines** | | |
| **free carnitine** | NS | NS |
| **C2-AC** | NS | NS |
| **C16-AC** | NS | NS |
| **C18:1-AC** | NS | NS |
| **C18:2-AC** | <0.05 | NS |
| **Saturated fatty acids** | | |
| **C20:0** | NS | NS |

| | | |
|---|---|---|
| **C22:0** | NS | NS |
| **C24:0** | NS | NS |
| **Monounsaturated fatty acids** | | |
| **C16:1Tn7** | NS | NS |
| **C18:1n7** | NS | NS |
| **C18:1n9** | NS | NS |
| **Polyunsaturated fatty acids** | | |
| **C18:2n6** | NS | NS |
| **C20:4n6** | NS | NS |
| **C20:5n3** | NS | NS |
| **C22:4n6** | NS | NS |
| **C22:5n3** | NS | NS |
| **C22:6n3** | NS | NS |

In the presence of significant interaction, the correlation between metabolite and NT-proBNP was tested in each group and the corresponding *P* values were noted. NS: not significant.

**Table S4. Interaction analysis with diabetic (type 2 diabetes) status**

| | **Diabetes** | |
|---|---|---|
| | noPH-HF | PH-HF |
| **Acylcarnitines** | | |
| **free carnitine** | NS | NS |
| **C2-AC** | NS | NS |
| **C16-AC** | NS | NS |
| **C18:1-AC** | NS | NS |
| **C18:2-AC** | NS | NS |
| **Saturated fatty acids** | | |
| **C20:0** | NS | NS |
| **C22:0** | NS | NS |

| | | |
|---|---|---|
| **C24:0** | NS | NS |
| **Monounsaturated fatty acids** | | |
| **C16:1Tn7** | NS | NS |
| **C18:1n7** | NS | NS |
| **C18:1n9** | NS | NS |
| **Polyunsaturated fatty acids** | | |
| **C18:2n6** | NS | NS |
| **C20:4n6** | NS | NS |
| **C20:5n3** | NS | NS |
| **C22:4n6** | NS | NS |
| **C22:5n3** | NS | NS |
| **C22:6n3** | NS | NS |

In the presence of significant interaction, the correlation between metabolite and NT-proBNP was tested in each group and the corresponding *P* values were noted. NS: not significant.

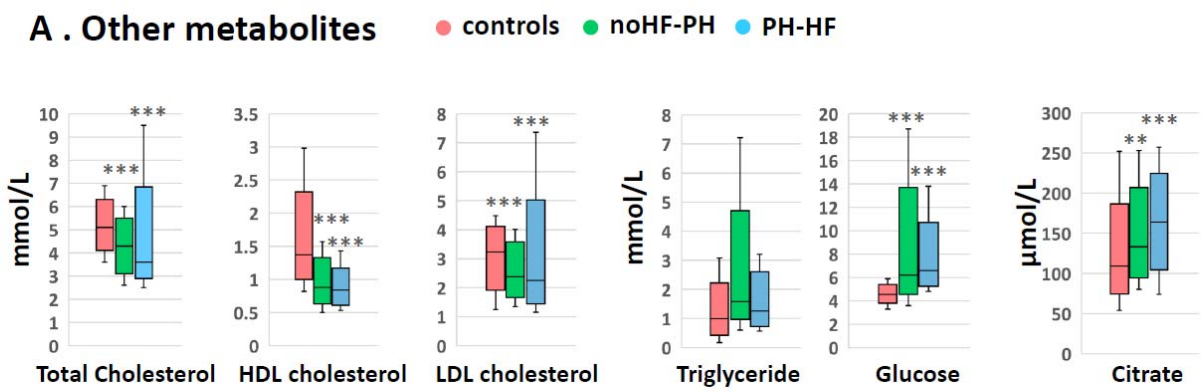


**Figure S1. Other metabolites that are differentially changed in PH-HF (N=33) and noPH-HF (N=27) compared to controls (N=72).**

Box plots depicting the most discriminant molecules from commercial biochemical analyses and citrate, which was measured using a MS-based approach. Comparison of noPH-HF (green) vs. controls (red) and PH-HF (blue) vs. controls. In the boxplots, rectangles represent the SD, the segment inside the rectangle the median and the whiskers above and below the maximum and minimum. *P < 0.05, **P < 0.01, ***P < 0.001 compared to controls.

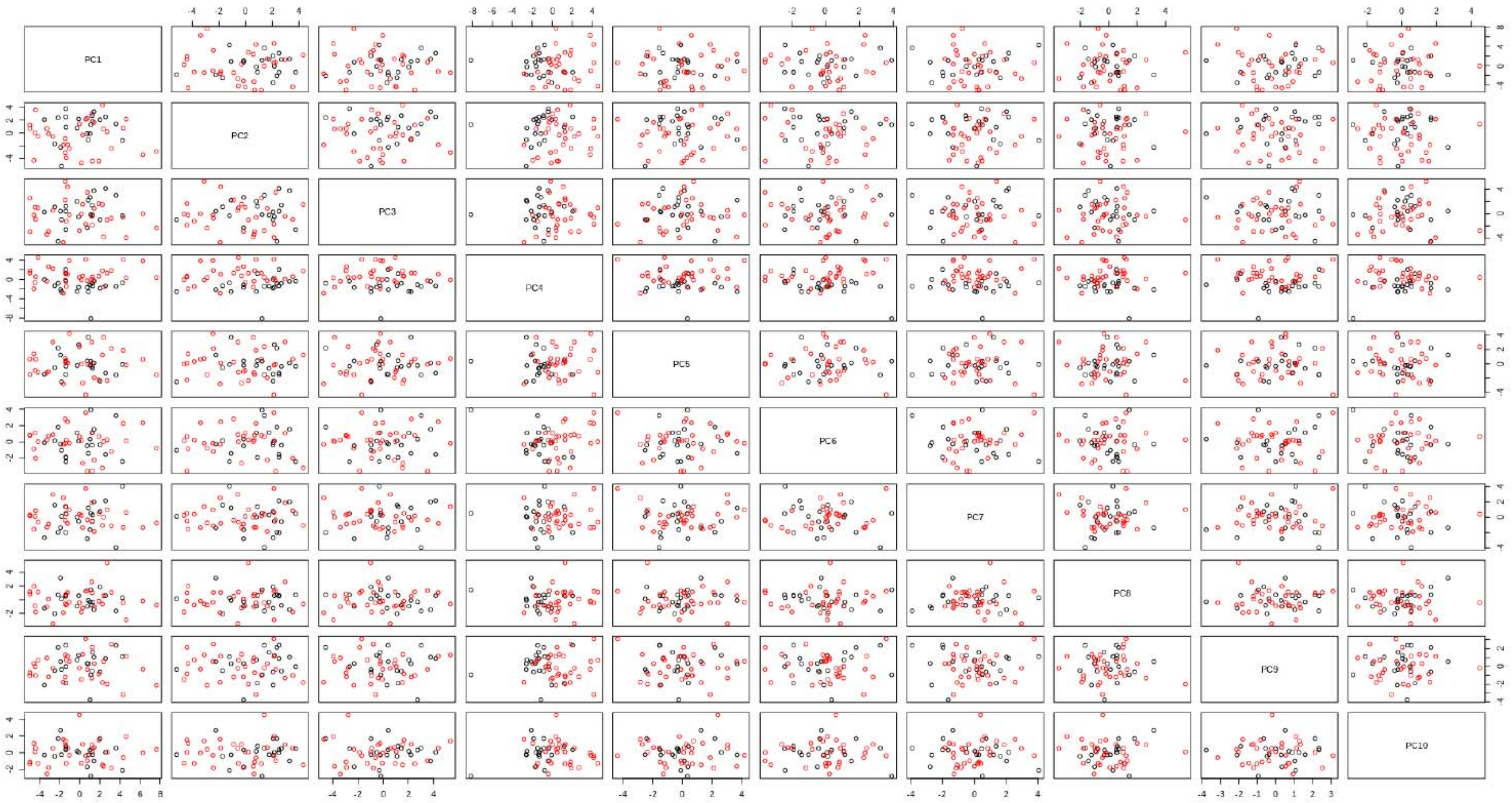


**Figure S2. PCA analysis reporting all the PC combination.** This whole PCA analysis was used to identify the better association and we observed a potential structure only in the presence of the PC4.

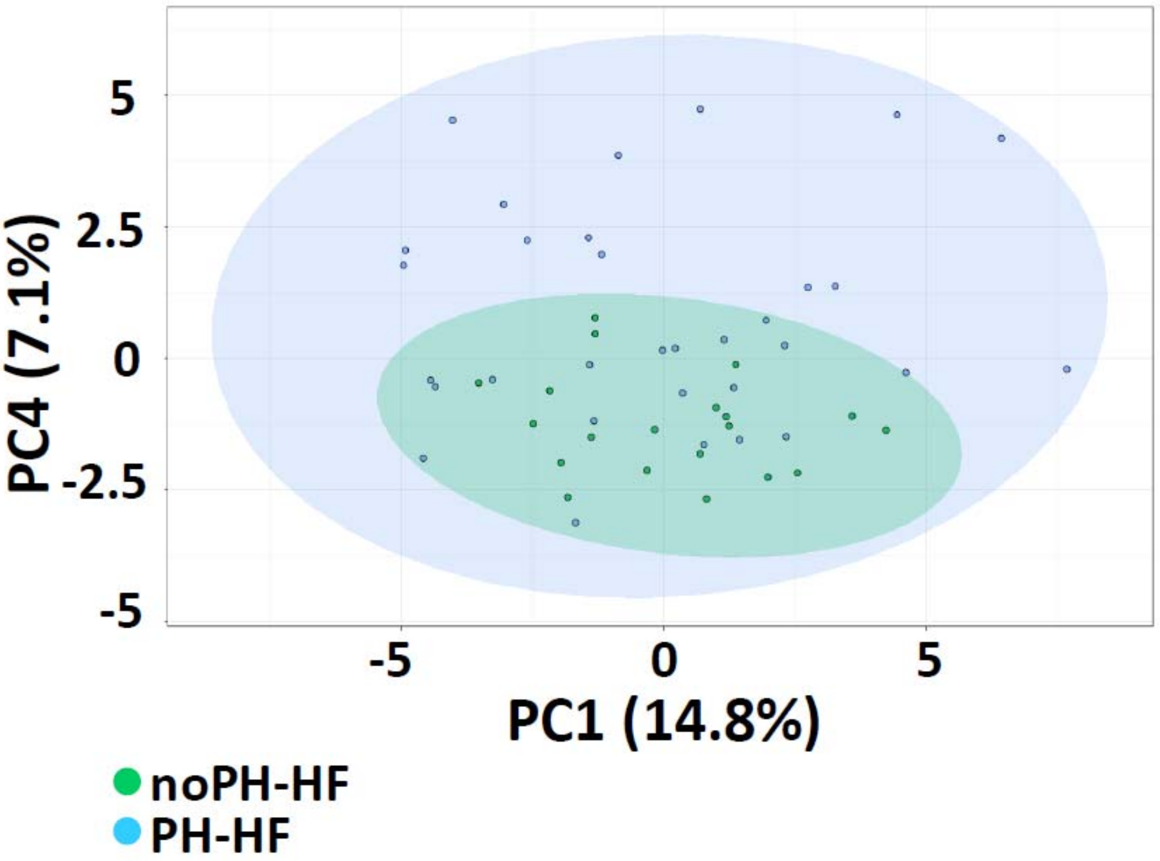


**Figure S3. Principal component analysis identified a higher heterogeneity in PH-HF (N=33) compared to noPH-HF (N=27).** A total of 55 variables were included in the analysis and comprised usual biochemical parameters as well as various metabolites measured by a combination of MS-based metabolomics approach targeting fatty acids, acyl-carnitines, organic acids and amino acids. Principal component 1 (PC1) and principal component 4 (PC4) accounted for 14.8% and 7.1% of the total variation, respectively. PH-HF were identified in blue and noPH-HF in green. Note that we excluded one outlier sample that was outside the confident ellipse.

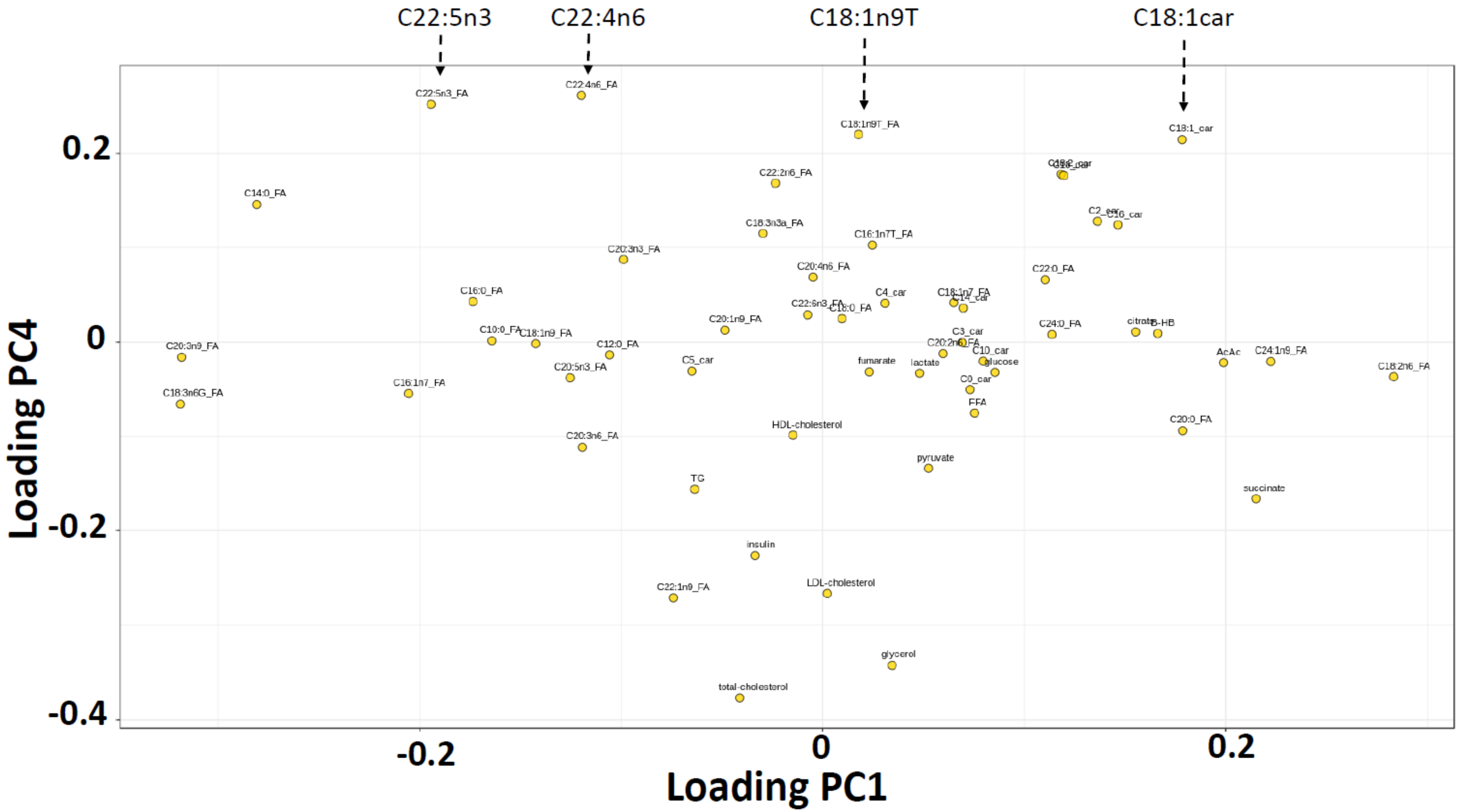


**Figure S4. Loading plot analysis showing the metabolites used for the PCA analysis.** We highlighted the 4 metabolites participating the most to the heterogeneity of the PH-HF group

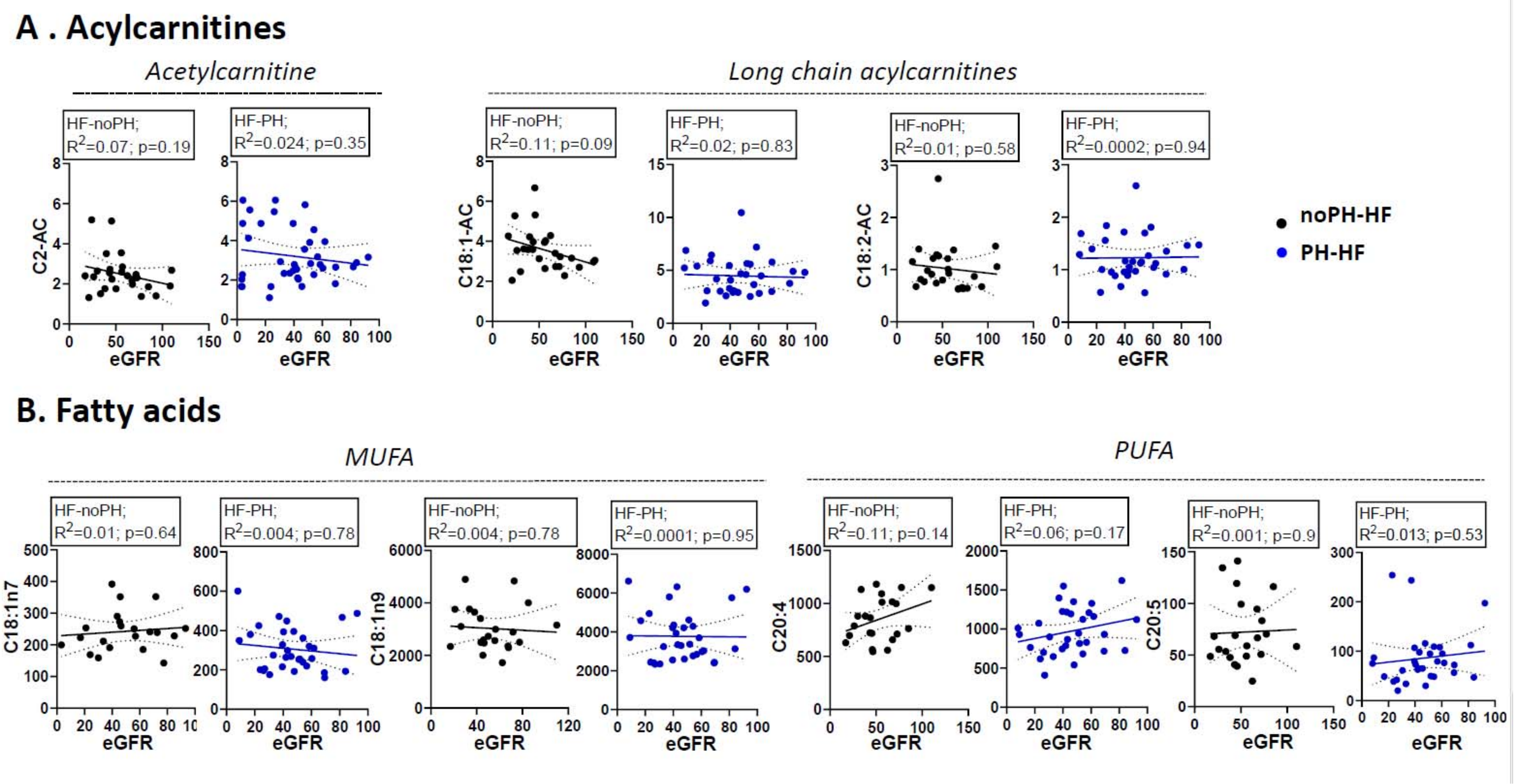


**Figure S5. Acylcarnitines and fatty acids do not correlate with eGFR in PH-HF and noPH-HF.** Pearson correlation analysis between eGFR and **(A)** acylcarnitines, **(B)** FA (MUFA and PUFA) in noPH-HF (black) or PH-HF (blue) patients. The dotted lines indicate the 95% confidence intervals.
